# The Use of Whey Powder to Improve Bread Quality: A Sustainable Solution for Utilizing Dairy By-Products

**DOI:** 10.3390/foods14162911

**Published:** 2025-08-21

**Authors:** Diana Fluerasu (Bălțatu), Christine Neagu, Sylvestre Dossa, Monica Negrea, Călin Jianu, Adina Berbecea, Daniela Stoin, Dacian Lalescu, Diana Brezovan, Liliana Cseh, Mariana Suba, Cătălin Ianasi, Ersilia Alexa

**Affiliations:** 1 Faculty of Food Engineering, University of Life Sciences “King Mihai I” from Timisoara, Aradului Street No. 119, 300645 Timisoara, Romania; fluerasu.diana@usvt.ro (D.F.); christine.neagu@usvt.ro (C.N.); dossasylvestre@usvt.ro (S.D.); monicanegrea@usvt.ro (M.N.); calinjianu@yahoo.com (C.J.); danielastoin@usvt.ro (D.S.); lalescu@usvt.ro (D.L.); 2 Food Science Research Center, University of Life Sciences “King Mihai I” from Timisoara, Aradului Street No. 119, 300645 Timisoara, Romania; 3 Faculty of Agriculture, University of Life Sciences “King Mihai I” from Timisoara, Aradului Street No. 119, 300645 Timisoara, Romania; adina_berbecea@usvt.ro; 4 Faculty of Veterinary Medicine, University of Life Sciences “King Mihai I” from Timisoara, Aradului Street No. 119, 300645 Timisoara, Romania; dianabrezovan@usvt.ro; 5 Romanian Academy, “Coriolan Dragulescu” Institute of Chemistry, Mihai Viteazu No. 24, 300223 Timișoara, Romania; lcseh@acad-icht.tm.edu.ro (L.C.); marianasuba@gmail.com (M.S.)

**Keywords:** composite flours, whey powder, wheat flour, bread, MIXOLAB, nutritional, sensory, physical–chemical properties

## Abstract

This paper aims to study the potential of whey, a by-product in the dairy industry, to be used as a sustainable and health-promoting ingredient in baking. In this regard, whey powder (WhF) was produced and incorporated into three composite flours consisting of wheat flour and whey powder in proportions of 5% (WhWF5), 10% (WhWF10), and 15% (WhWF15). These composite flours were then used to produce bread. The nutritional properties (proximate composition, macro and microelement content) and bioactive compounds (total polyphenols and antioxidant activity) were assessed for both the composite flours and the resulting breads. In addition, the rheological behavior of the dough was evaluated using the Mixolab system, while the microstructural characteristics and physical properties of the composite flours were analyzed using Small/Wide Angle X-ray Scattering (SAXS/WAXS) and Fourier Transform Infrared Spectroscopy (FTIR). Sensory evaluation of the breads was also performed. The results demonstrated a positive effect of the whey powder addition on the nutritional profile of both composite flours and bakery products, particularly through increased protein levels (25.24–37.77% in fortified flours vs. 11.26% in control; 16.64–18.89% in fortified breads vs. 14.12% in control) and enhanced mineral content (11.27–80.45% higher compared to white wheat bread), alongside a reduction in carbohydrate content. Bread fortified with 15% whey powder showed higher monolement with increases of 27.80% for K, 7.01% for Mg, and 28.67% for Ca compared to control bread without whey. The analysis of the Mixolab charts confirmed the progressive influence of whey powder on dough rheology. While water absorption remains high, other functional parameters, such as gluten quality, kneading capacity, and starch viscosity, were negatively affected. Nonetheless, the nutritional advantages and reduced retrogradation tendency may offset these drawbacks in the context of developing functional bakery products. Formulations containing 5–10% whey powder appear to offer an optimal balance between technological performance, nutritional quality, and sensory acceptance.

## 1. Introduction

Whey is a valuable by-product of the dairy industry, obtained during the processing of milk for cheese production. Traditionally regarded as industrial waste, recent research has highlighted its potential for valorization in various food industries, particularly in baking, beverages, jam production, and as a component in soup powder [1]. Whey is widely incorporated into functional products, such as nutrition supplements for the elderly and sports, due to its rich nutritional composition, which includes high biological value proteins, lactose, vitamins (especially B2 and B12), minerals (notably calcium and phosphorus), and various enzymes [2]. This composition makes whey a functional ingredient with multiple technological and nutritional applications [1,2]. Typically, whey contains approximately 0.6–0.8% proteins, 4.5–5% lactose, 0.5–0.6% minerals, and a low-fat content [3]. Whey proteins (α-lactalbumin, β-lactoglobulin, and immunoglobulins) possess important functional properties, including excellent solubility, emulsifying and foaming capacity, as well as antioxidant and antimicrobial activity [4]. These characteristics make them suitable for use in processed foods, including baked goods. The use of whey in baking has been explored both as a partial or total complete replacement for water or milk in dough formulation [1], and it can be added in liquid, concentrated, or powdered form [3,5,6]. Incorporating fermented whey into bread formulations did not significantly affect key technological parameters, such as specific volume, water activity, crust color, or crumb alveolar structure, while extending the product’s shelf life compared to conventional bread [7,8]. Studies on pan bread prepared with dried whey protein concentrate have indicated that a concentration up to 20% reduces hardness compared to the control [9]. The addition of whey or whey protein concentrates can also increase the protein content of the final product, improve crispness, enhance crumb structure, and slow staling, thereby prolonging shelf-life and bread flavor. Furthermore, whey enhances water retention, resulting in a softer texture and longer freshness [10]. Lactose in whey acts as a fermentable substrate for yeast, promoting fermentation and leading to improved crust caramelization and flavor [11].

In bread production, the formation of the gluten network is crucial for trapping fermentation gases and achieving an airy crumb structure. Whey proteins, especially β-lactoglobulin and α-lactalbumin, differ structurally from the glutenins and gliadins in wheat flour, and their addition can alter dough rheology [12]. At low inclusion levels (below 10%), whey proteins may interfere with the gluten network by competing for water and through electrostatic interactions, weakening its structure and reducing gas retention capacity [7]. However, whey proteins also have high hydration and gel-forming capacity when heated, which, at higher addition levels (10–15%), can enhance elasticity and crumb cohesion [12]. Therefore, depending on the dosage, whey powder can exert both negative and positive effects on the rheological and structural properties of dough.

The incorporation of whey in baking supports the development of functional products (e.g., high-protein for athletes) and aligns with circular economy principles by valorizing a secondary stream that might otherwise have a negative environmental impact [13]. Whey also offers a cost-effective means of improving the nutritional quality of bread by replacing more expensive ingredients such as eggs or milk powder. For example, adding 10% sweet whey to white bread has been shown to increase loaf volume and improve sensory scores in taste tests [14]. Other research indicates that whey can be incorporated at levels up to 20% in multigrain bread without adversely affecting baking performance, while enhancing crust color through improved Maillard browning and increasing the total solids content. Although crumb texture remained unaffected, likely due to high baking temperatures (above 160 °C) and the 15% total solids, crust browning and flavor were improved [15].

The whey from milk is a valuable resource for baking, offering both technological and sensory benefits in addition to nutritional improvements [16]. Sensory evaluations of composite sorghum–rice gluten-free bread fortified with whey showed no negative impact on organoleptic properties, and in fact, overall acceptability was higher than that of commercial breads [17]. Collectively, these findings confirm that incorporating whey into bread and pastry recipes facilitates the development of sustainable, innovative food products in line with the modern market demands [16,17].

In this context, the present study aims to explore the potential of incorporating whey powder at various levels into bread recipes and to evaluate the nutritional, functional, and rheological properties of the composite flours and bread based on wheat flour with added whey. The technological advantages and innovation approach of this study lie in the use of whey powder, obtained according to the method described herein, which offers a more accessible solution for processors compared to whey protein concentrate or isolate, which requires advanced processing.

The originality of this research is reflected in its comprehensive and in-depth approach, addressing nutritional, sensory, functional, technological, and rheological aspects to determine optimal technological solutions for technology transfer. The core research problem addressed is how to improve the nutritional, technological, and functional attributes of bread through the inclusion of whey powder, without compromising sensory quality or consumer acceptance. This topic holds practical relevance in light of current trends toward the utilization of the food industry within the framework of the circular economy.

## 2. Materials and Methods

### 2.1. Materials

In order to obtain whey powder used for the fortified flours, cow’s milk whey was sourced from BRO-LACT FARM, a dairy factory located in Mehedinți, Romania.

White wheat flour type 550 (WF) was supplied by the BANEASA Factory, Romania. This, with a mineral content of 0.55%, was selected as the control, as it is the standard baking flour used for white bread production in Romania.

For bread preparation, the following ingredients were used: type 550 white flour, composite flours, salt, yeast, sugar, and water. All ingredients were purchased from local markets.

### 2.2. Obtainment of Whey Powder

**Figure 1:**
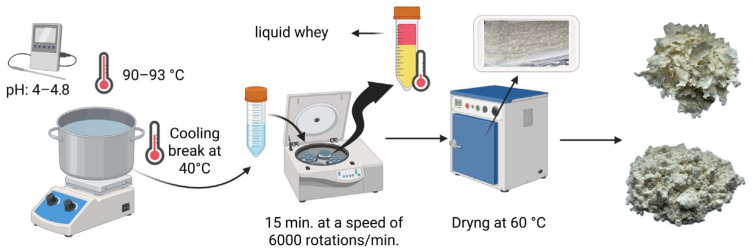
The obtainment of whey powder (Figure created in Biorender.com).

To obtain the whey powder, 16 L of whey with a pH of 4.5 was heated to a temperature of 92 °C using a household stove (Selecline, Shenzhen, China) and maintained at this temperature for 3 min, followed by a cooling phase down to 40 °C. The whey was transferred to 50 mL flasks and placed in a centrifuge (Universal 320R, Hettich Lab Technology, Tuttlingen, Germany) and centrifuged for 15 min at a speed of 6000 rotations/minute, at a temperature of 4 °C. The resulting precipitate was removed and placed to dry in an oven (Air Concept Precisa, Kruszwica, Poland) for 6 h at a temperature of 60 °C. After drying, the whey powder was obtained, which was ground in a grinder (Silvercrest SKMS 150 A1, Kompernass Handels GmbH, Bochum, Germany), thus obtaining a fine, white powder (Figure 1).

### 2.3. Composite Flours Preparation

**Figure 2:**
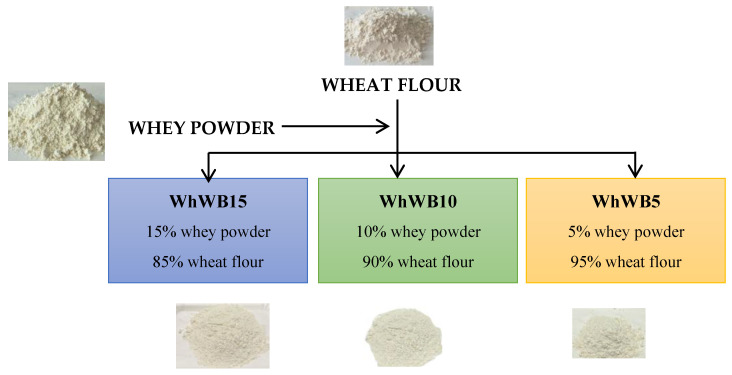
Composite flours based on wheat flour and whey powder.

Three mixtures were prepared from wheat flour with whey powder (WhF): WhWF5—5% whey powder, 95% wheat white flour, WhWF10—10% whey powder, 90% wheat white flour, and WhWF15—15% whey powder, 85% wheat white flour (Figure 2).

### 2.4. Fortified Bread Preparation

Three experimental composite breads were prepared:WhWB5—containing 5% whey powder, WhWB10—containing 10% whey powder, WhWB15—containing 15% whey powder, and a control sample containing 100% white flour (Table 1).

**Figure 3:**
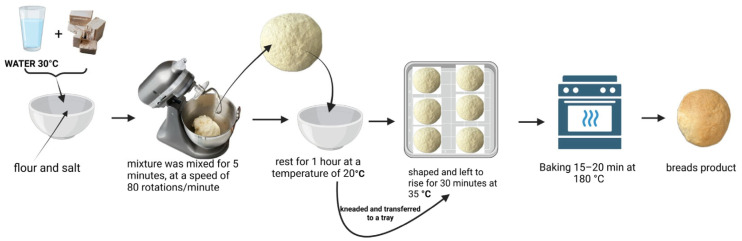
The technological process of fortified breads with whey powder (Figure created in Biorender.com).

The bread preparation process was carried out as follows: yeast was first hydrated with water at 30 °C in a mixing bowl, followed by the addition of composite flours and salt. The ingredients were mixed for 5 min using a spiral hook mixer (Revolution, Hendi Polska, Godki, Poland) at a speed of 80 revolutions per minute. The resulting dough was transferred to a stainless steel bowl and rested for approximately 1 h at 20 °C. It was then kneaded and placed on a tray for fermentation at 35 °C for 30 min. Subsequently, the dough was baked in a preheated oven at 230 °C for 25 min. After baking, the bread was allowed to cool at room temperature for 24 h (Figure 3).

### 2.5. Determination of the Proximate Composition of Composite Flours and Breads

The proximate composition was determined using standard AOAC methods [18,19,20]. Ash content was analyzed following ISO Method No. 2171:2007 [18], while moisture and protein were assessed according to ICC standard methods and AOAC guidelines [19]. Fat content was measured using the AOAC Method 920.39 [20]. Carbohydrate content (g/100 g) was calculated following the procedure described by Dossa et al. [21], using the following equation:

*Carbohydrate* (g/100 g) = 100 − (*fat* + *protein* + *water* + *ash*) (1)

### 2.6. Determination of Macro and Microelements

The macro- and microelements content was determined by atomic absorption spectroscopy (AAS) using the Varian 220 FAA equipment (Palo Alto, CA, USA) [22]. Briefly, the ash sample was obtained at point 2.4, which was treated with 10 mL of 20% hydrochloric acid (Chimreactiv, Bucharest, Romania). The solutions obtained were analyzed individually by AAS, with each element being measured at its specific wavelength, using cathode lamps for the following analyzed elements: K, Ca, Mg, Na, Fe, Zn, Cu, Mn, Ni, and Cr. The instrument was calibrated before each series of analyses with reference standard solutions, and the results were expressed in mg/kg of dry sample.

### 2.7. Determination of Physical–Chemical Properties of Bread

#### 2.7.1. Determination of Bread Volume

Bread volume was determined using the classic FORNET apparatus (Chopin Technologies, Paris, France), which operates on the principle of volumetric seed displacement. Before measurement, baked and completely cooled bread (at room temperature for at least 2 h) was placed in the measuring chamber of the apparatus. The apparatus is equipped with a calibrated container, into which a known amount of seeds (usually millet) is poured, up to a marked reference level. After placing the bread in the container, the seeds are allowed to flow gravitationally around the sample, occupying the free spaces. Once the bread is completely surrounded, the new level of seeds is recorded, and the difference in volume between the initial and final levels corresponds to the bread volume. The result is expressed in cubic centimeters (cm^3^). For accuracy, the determination was performed in triplicate, and the average of the values obtained was reported [23].

The bread volume is calculated with the following formula:

*Volume* (*V*) = (*V*1/*m*) × 100 (2)

where:

V1—volume of displaced rapeseeds, in cm^3^;
m—mass of the bread sample, in g.

#### 2.7.2. Determination of Bread Elasticity

The bread elasticity was determined by pressing in a cylinder form [24] and calculated with the following formula:

*Elasticity* (*E*) = (*B*/*A*) × 100 (3)

where:

A—height of the core cylinder before pressing, in cm;
B—height of the core cylinder after pressing and its return to the initial position, in cm.

#### 2.7.3. Determination of Bread Porosity

The method is based on determining the specific mass of the pore-free core [24] and the porosity is calculated with the following formula:

*Porosity* = [*V* − (*m*/*ρ*)/*V*] × 100 (4)

where:

V—the volume of the core cylinder, in cm^3^;
m—the mass of the core cylinder, in g;
*ρ*—the density of the compact core, in g/cm^3^.

#### 2.7.4. Determination of the H/D Ratio

The product height and diameter are measured, and their ratio is calculated with the following formula [25]:

*Height*/*diameter* *ratio* = *H*/*D* (5)

where:

H—height of the product, in cm;
D—arithmetic mean of two perpendicular diameters, in cm.

### 2.8. Determination of the Phytochemical Profile

#### 2.8.1. Preparation of Alcoholic Extracts

For WF, WhF, and each composite flour and bread sample, 1 g was accurately weighed into sealed containers, followed by the addition of 10 mL of 70% (*v*/*v*) ethanol (Chimreactiv, Bucharest, Romania). The containers were securely closed, and the mixtures were stirred using a magnetic stirrer (IDL, Freising, Germany) for 30 min to ensure proper extraction. After stirring, the samples were filtered through Whatman No. 1 filter paper, and the resulting filtrates were collected. These ethanolic extracts were subsequently used for the determination of total polyphenol content and total antioxidant activity using the DPPH radical scavenging assay.

#### 2.8.2. Evaluation of the Total Phenolic Content (TPC)

The total phenolic content of WF, WhF, composite flours, and bread samples containing different concentrations of whey powder was determined using the Folin–Ciocalteu method [26]. Briefly, 0.5 mL of the prepared extract was mixed with Folin–Ciocalteu reagent (diluted 1:10) (Sigma-Aldrich Chemie GmbH, Munich, Germany) and 1 mL of sodium carbonate solution (60 g/L) (Geyer GmbH, Renningen, Germany). The mixture was then incubated at 50 °C for 30 min in an INB500 incubator (Memmert GmbH, Schwabach, Germany). After incubation, the absorbance was measured at 750 nm using a Specord 205 UV–Vis spectrophotometer (Analytik Jena AG, Jena, Germany). The results were expressed as milligrams of gallic acid equivalents (GAE) per 100 g of sample.

#### 2.8.3. Antioxidant Activity

The antioxidant activity of WF, WhF, composite flours, and fortified breads was evaluated using the 2,2-diphenyl-1-picrylhydrazyl (DPPH) radical scavenging assay (Sigma-Aldrich; Merck KGaA, Darmstadt, Germany) [26]. The reaction mixture consisted of 1 mL of diluted extract (1:10) and 2.5 mL of a 0.03 mM DPPH solution. The samples were incubated for 30 min at room temperature, protected from light to prevent degradation of the DPPH radical. Following incubation, the absorbance was measured at 518 nm using a UV–Vis spectrophotometer (Specord 205; Analytik Jena AG, Jena, Germany). Ethanol was used as the positive control in the assay. The antioxidant activity was calculated using the following equation:

*RSA* (%) = (*Acontrol* − *Asamples*/*Acontrol*) × 100 (6)

where:

Acontrol—Absorbance of control sample denotes the control absorbance values, and Asamples—Absorbance of samples.

### 2.9. Rheological Analysis

Rheological analysis was conducted to evaluate the effect of replacing wheat flour with whey powder flour (5%, 10%, and 15%) on the rheological properties using Chopin Mixolab equipment (Chopin Technologies, Paris, France) and the “Chopin+” protocol [27]. Quantities ranging from 42 to 50 g of the sample (depending on the sample’s moisture content) were placed in the Mixolab bowl and mixed. After tempering the solids, water was added to achieve optimal consistency. Particular attention was paid to determining water absorption to ensure the complete hydration of all the components.

The parameters taken into consideration from the Mixolab profile were water absorp- tion, dough development time, stability (mixing resistance of dough), maximum torque during mixing—C1, weakening of the protein—C2, which manifests as a result of mechanical stress as the temperature rises, the rate of starch gelatinization—C3, minimum torque—C4, and torque—C5 after cooling at 50 °C. In addition, Mixolab determined the following parameters: cooking stability (C4/C3), protein weakening under a heating ef- fect (alpha slope), starch gelatinization speed (beta slope), enzyme degradation speed (gamma slope), and starch retrogradation at the cooling stage (C5–C4), which represents the shelf-life of the final products [27].

### 2.10. Sensory Evaluation

Four samples of bread made from wheat flour and different percentages of whey powder (WhWB5—white bread with 5% whey; WhWB10—white bread with 10% whey; and WhWB15—white bread with 15% whey) were evaluated by a panel of 20 evaluators (9 men and 11 women) who are non-smokers with no known food allergies. A control sample (WB), consisting of bread made from white wheat flour, was also evaluated together with the other bread samples. All panelists are students of the Faculty of Food Engineering, who have attended Sensory analysis courses. The evaluators signed a consent form before the actual tasting of the products, emphasizing that they had been informed of the purpose and nature of the activity, they understood that their participation was voluntary, that they could withdraw at any time without consequences, and that they took full responsibility for the consumption of the samples provided during this activity.

The 1-cm-thick slices of bread, with crust, were presented to the assessors on paper plates, coded with two-digit characters and served in random order, under normal lighting conditions and at room temperature. Panelists were asked to rate the sensory attributes (appearance, taste/flavor, texture/porosity, taste/chewiness, and overall acceptability) using a five-point hedonic scale [28], with the following graduation: 1 = Strongly Disagree; 2 = Slightly Disagree; 3 = Neutral; 4 = Slightly Agree; 5 = Strongly Agree [28].

### 2.11. The Microscopic Analysis of Composite Flours

The microscopic examination of composite flours, control, and whey powder was performed using a microscope Olympus CX-41 (Olympus Corporation, Tokyo, Japan), using 40× objectives. A small amount of sample was taken (e.g., a pinch of flour) and was placed on a clean glass slide, and a drop of Lugol solution (1% iodine, 2% potassium iodide) was added on top. The preparation was covered with a coverslip, avoiding the formation of air bubbles, and the images were observed by the microscope. After being stained with iodine, starch takes on a deep blue-purple color—a specific reaction.

### 2.12. Fourier Transform Infrared Spectroscopy (FTIR) of Composite Flours

For FTIR analysis, Nicolet Is50 FT-IR (Thermo Fisher Scientific, Waltham, MA, USA) equipment, equipped with an ATR crystal, was used.

FTIR-ATR spectroscopy, or Attenuated Total Reflectance Fourier Transform Infrared spectroscopy, was performed at room temperature by using a Nicolet™ iS50 FTIR Spectrometer. The IR spectra were obtained in the spectral range of 4000–400 cm^−1^, 32 scans at 4 cm^−1^ resolution. The procedure started with the pre-drying of the sample, by keeping it in an oven at 40 °C overnight, in order to remove moisture that could interfere with the spectroscopic analysis. After complete drying, the sample was placed directly on the ATR crystal of the FTIR spectrometer.

### 2.13. Small-Wide Angle X-Ray Scattering (SAXS/WAXS) of Composite Flours

The Xenocs Xeuss 3.0 (Xenocs SAS, Grenoble, France) was used for the determination of powder samples. The device uses Cu (Genix 3D) as a source and a Dectris Eiger2 Si 1M detector. All analyses are performed in a vacuum at room temperature and using Kapton film. For the SAXS and WAXS measurements, a distance of 1800 mm and 45 mm with HFlux collimation for a time of 300 s was used. The obtained results were interpreted and fitted with the XSACT 2.10 program:Xenocs, “XSACT: X-ray Scattering Analysis and Calculation Tool.” xsact.xenocs.com, 2023. SAXS and WAXS data analysis software—Version 2.10.

The fitting for the SAXS region was performed in the Guinier region between 0.01 and 0.1 A^−1^, taking into account that the particles are spherical and using the following equation:

I(q) = exp(-qRg^2^/3). (7)

where:

I(q)—scattered intensity;
q—scattering variable;
Rg—radius of gyration.

### 2.14. Statistical Analysis

All determinations were performed in triplicate. The results are presented as the mean values ± standard deviation (SD). The differences between the mean values were analyzed using Duncan’s Multiple Range Test after ANOVA. Significant differences were considered when the *p*-values were less than 0.05. All statistical analyses were performed using R Statistical Software (v4.3.3; R Core Team 2023, Viena, Austria).

## 3. Results and Discussion

### 3.1. Proximate Composition of Composite Flours and Fortified Breads with Whey Powder

**Figure 4:**
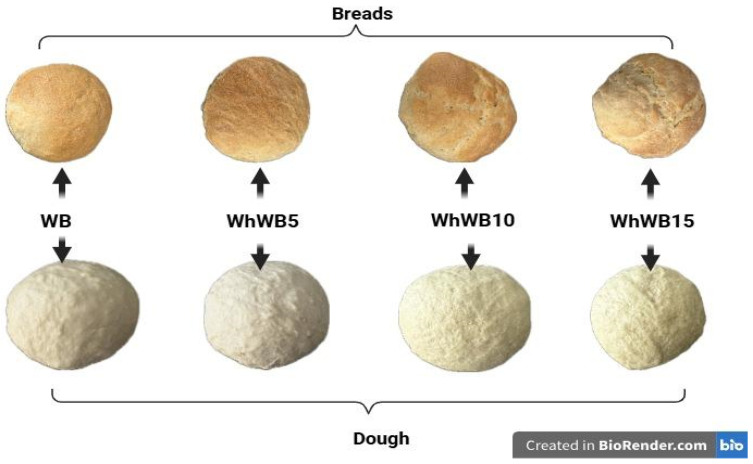
The obtainment of fortified breads with whey powder.

The fortified bread obtained based on composite flours is presented in Figure 4, while the proximate composition of composite flours and fortified breads with whey powder is presented in Table 2.

The data obtained from the nutritional analysis of composite flour and baked products highlight significant changes in the chemical parameters with the increase in the proportion of the WhF ingredient in the recipe. Regarding moisture, a slight decrease was observed both in flour samples (from 12.70% in the control WF flour to 12.15% in the WhWF15 variant) and in baked products (from 29.37% in the control WB bread to 27.03% in WhWB15). This result can be associated with the influence of WhF on water retention capacity, a phenomenon also reported in the scientific literature in the context of using alternative protein ingredients that can modify gluten structure and dough rheology [29,30].

The ash content has significantly increased in all variants enriched with WhF. High values were recorded in whey powder (3.01%) compared to the WF control (0.59%), and in the case of breads, an increase was observed from 1.33% (WB) to 2.40% (WhWB15). Supplementing bread with whey powder in a proportion of 5–15% leads to an increase in mineral substances, between 11.27 and 80.45% compared to white bread made from wheat flour, which argues the nutritional potential of whey. This trend reflects a significant mineral enrichment of the products, confirming that the WhF ingredient is a valuable source of micronutrients. These findings are supported by previous studies highlighting the potential of unconventional flours, such as those obtained from insects, legumes, or pseudocereals, in increasing the mineral intake in widely consumed foods [31].

The protein content significantly increased with the addition of WhF. Flour enriched with 15% WhF (WhWF15) showed a protein content of 25.24%, compared to 11.26% in WF, while in bread, the values varied from 14.12% (WB) to 18.89% (WhWB15). The nutritional profile of composite flours, in terms of protein content, increased by 3.35 times with the addition of 15% WhF, respectively, with 33.21% for the bread with an addition of 15% WhF compared to the control: WF and bread made only from wheat flour. These increases demonstrate the effectiveness of adding WhF in improving the protein value of products, aligning with other studies that support the use of alternative protein sources in combating malnutrition and diversifying diets [32,33].

Regarding lipid content, a slight decrease was observed in the flour mixtures (from 1.35% in WF to 0.76% in WhWF15) and in bread varied from 6.68% in WF to 3.47% in WhWF15. The fluctuations can be explained by the interaction of proteins with fats during the technological process, a topic also discussed in the specialized literature [34].

The carbohydrate content progressively decreased in the samples with the addition of WhF, from 74.11% (WF) to 49.10% (WhWF15) in composite flours. This result is explained by the partial substitution of the carbohydrate fraction with protein and mineral fractions derived from the functional ingredient, a common aspect observed in the literature when replacing wheat flour with alternative ingredients rich in proteins [35].

### 3.2. Macro and Microelements Content of Composite Flours and Breads Fortified with Whey Powder

The macro- and microelements content of composite flours and fortified breads with whey powder is presented in Table 3.

The results obtained highlight a significant improvement in nutritional values following the addition of whey concentrate, particularly regarding the content of macroelements. The potassium (K) content showed a considerable increase in the whey mixtures, from 1388.26 mg/kg in wheat flour (WF) to 1906.19 mg/kg in flour with 15% whey (WhWF15). Similarly, bread made from enriched flour showed increased values, reaching up to 1542.59 mg/kg (WhWB15), compared to 1207.06 mg/kg in the control bread. This trend is in line with data from the literature, which indicates a high concentration of potassium in sweet whey (approximately 1500 mg/L) [36].

Calcium (Ca) followed a similar pattern, increasing from 337.58 mg/kg (WF) to 544.78 mg/kg (WhWF15). This increase is supported by data on the mineral composition of whey, which naturally contains calcium in a significant proportion (216–358 mg/kg) [37]. The increase in the Ca content in bread fortified with WhF is between 22.36 and 28.67% compared to white bread without additives, thus suggesting the important nutritional contribution of whey powder.

Regarding magnesium (Mg), a progressive increase was noted from 532.5 mg/kg in WF to 702.84 mg/kg in WhWF15. Whey, known for its moderate Mg content, thus contributes to the enrichment of cereal products. Additionally, the sodium (Na) content has steadily increased, especially in the enriched breads (for example, 209.18 mg/kg in WB vs. 222.85 mg/kg in WhWB15), which reflects both the contribution of whey and the possible addition of salt in the bread recipe.

The copper (Cu) content significantly increased depending on the percentage of whey added, from 2.64 mg/kg in WF to 5.41 mg/kg in WhWF15. This can be explained by the presence of copper in whey, especially in the form bound to proteins [37]. In fortified bread, the increase in Cu content varied between 56.58 and 88.15% depending on the percentage of whey powder added to the bread.

Zinc (Zn) and iron (Fe) exhibited less consistent variations. Although iron showed a slight increase in sample WhWF15 (14.23 mg/kg) compared to WF (11.34 mg/kg), zinc had a fluctuating development without a clear trend. Manganese (Mn) content remained relatively constant across all samples, while nickel (Ni) values increased proportionally with the addition of whey, from 0.88 mg/kg (WF) to 2.83 mg/kg (WhWF15). Chromium (Cr), on the other hand, was not significantly influenced by the addition of whey, with values ranging between 0.60 and 0.75 mg/kg.

These results are consistent with previous findings on the mineral composition of whey, which highlight its richness in micronutrients [37].

A comparison between raw flours and baked breads shows that the baking process leads to slight decreases in certain elements (such as Mn and Zn), likely due to losses from volatilization or thermal transformations. However, most macroelements remain stable, suggesting good retention of minerals after thermal treatment. In conclusion, the obtained data confirm that the addition of whey concentrate to wheat flour contributes to improving the mineral profile of baked goods, particularly through a significant increase in the content of potassium, calcium, magnesium, and copper. These results are supported by the specialized literature on the mineral composition of whey and can serve as a valuable argument for its use in fortifying widely consumed foods [37].

### 3.3. Physical–Chemical Properties of Fortified Breads with Whey Powder

The breads obtained were subjected to physical–chemical analyses: volume, porosity, elasticity, and the height/diameter (H/D) ratio (Table 4).

The determination of the physical indices of bread samples obtained from wheat flour (control, WB) and from wheat flour with added whey powder in proportions of 5%, 10%, and 15% (WhWB5, WhWB10, WhWB15) highlights the significant influence of this ingredient on the quality characteristics of the final product.

The porosity of the bread decreased progressively with the increase in the percentage of added whey: from 77.87% in the control sample to 70.91% in the sample with 15% whey. This behavior can be attributed to the changes experienced by the gluten network, whose gas retention properties are affected by the presence of proteins and salts from whey [38,39]. A reduction in porosity has also been reported in other studies that used whey proteins in baking formulas, which is correlated with a higher crumb density and a more compact texture. The study of Amina et al. (2021) highlighted that the values of dough overpressure or tenacity (P), dough extensibility (L), swelling index (G), baking strength (W), elasticity index, and H/D ratio decrease with the increase in whey content in bread [40].

The core elasticity had an uneven evolution. The control sample recorded a value of 90%, while the samples with additions of 5% and 10% whey showed a decrease to 86.53% and 82.96%, respectively. Surprisingly, with a 15% addition, elasticity increased to 91.81%, even exceeding the control value. This result could be explained by a potentially more stable protein network formed at higher whey concentrations, where complex protein interactions and increased hydration could contribute to better structure retention during baking [41]. It is also possible that certain peptides from whey contribute positively to the extensibility of the dough at higher addition levels [42].

The H/D ratio is an indicator of the vertical development of bread and the retention of volume during baking. The control sample recorded a ratio of 0.945, while the one with 5% whey showed a decrease (0.885). However, the WhWB10 and WhWB15 samples had higher values than the control (0.965 and 0.966). These results suggest that at higher concentrations, whey may help maintain the shape of the bread, likely by strengthening the protein network and improving the rheology of the dough [43]. The literature has highlighted that protein additions can influence the height and stability of breads, depending on their interaction with gluten and their water retention capacity [44].

### 3.4. Phytochemical Profile of Fortified Flours and Breads with Whey Powder

Flour and bread samples were subjected to phytochemical analyses, namely, the total polyphenol content (mg/100 g) and antioxidant activity (%) (Table 5).

The determination of the total polyphenol content and the evaluation of antioxidant activity using the DPPH method in composite flour samples and in finished bakery products highlighted significant variations depending on the proportion of the functional ingredient WhF. The total polyphenol content significantly increased with the increasing percentage of WhF in the flour mixtures. Thus, values varied from 184.98 mg/100 g in the control flour (WF) to 323.38 mg/100 g in the variant with 15% WhF (WhWF15), with a notable peak in the case of pure WhF (532.56 mg/100 g). The same trend was observed in bakery products, where the control bread (WB) had 229.29 mg/100 g, while the bread enriched with 15% WhF (WhWB15) reached 348.53 mg/100 g. These results indicate that WhF is a rich source of phenolic compounds, with significant bioactive potential. Studies from the literature confirm that the addition of unconventional ingredients such as plant extracts, legumes, or even processed animal sources (such as edible insects) contributes to the increase in the total polyphenol content in food products [45,46]. The addition of whey powder led to a significant increase in the total phenolic content of the bread, likely due to the presence of bioactive peptides and amino acids in whey that can interact with phenolic compounds and enhance their extractability, as also reported by Dobhal et al. (2024) [13].

However, the antioxidant activity determined by the DPPH method did not increase in parallel with the polyphenol content. In fact, the DPPH (%) values slightly decreased with the increase in the addition of WhF. In the flour samples, the antioxidant activity decreased from 28.26% (WF) to 25.57% (WhWF15), and in the breads from 27.25% (WB) to 24.18% (WhWB15). This discrepancy can be explained by the nature of the phenolic compounds present in WhF, which, although they contribute to the total polyphenols, may have a lower antioxidant capacity compared to the native polyphenols from wheat. Furthermore, the baking technological process may contribute to the partial degradation of antioxidant compounds or to the alteration of their chemical structure, thus reducing their effectiveness in free radical scavenging reactions [47,48]. Interestingly, the breads enriched with WhF still exhibited higher values of polyphenol content compared to the control, indicating good stability of these compounds during thermal processing. This observation is consistent with other studies that show that polyphenols associated with protein or lipid matrices can be partially protected against thermal degradation [49].

The results obtained demonstrate that the addition of the WhF ingredient significantly enriches bakery products with polyphenols, thus contributing to their functional value. However, the antioxidant efficiency determined by the DPPH test did not increase proportionally, which suggests that the biological activity of the present compounds depends not only on quantity but also on their type and stability during processing.

### 3.5. Analysis of Correlation Between Physical–Chemical, Macro and Microelements, and Phytochemical Parameters in Composite Flours with Whey Powder and in Fortified Bread

**Figure 5:**
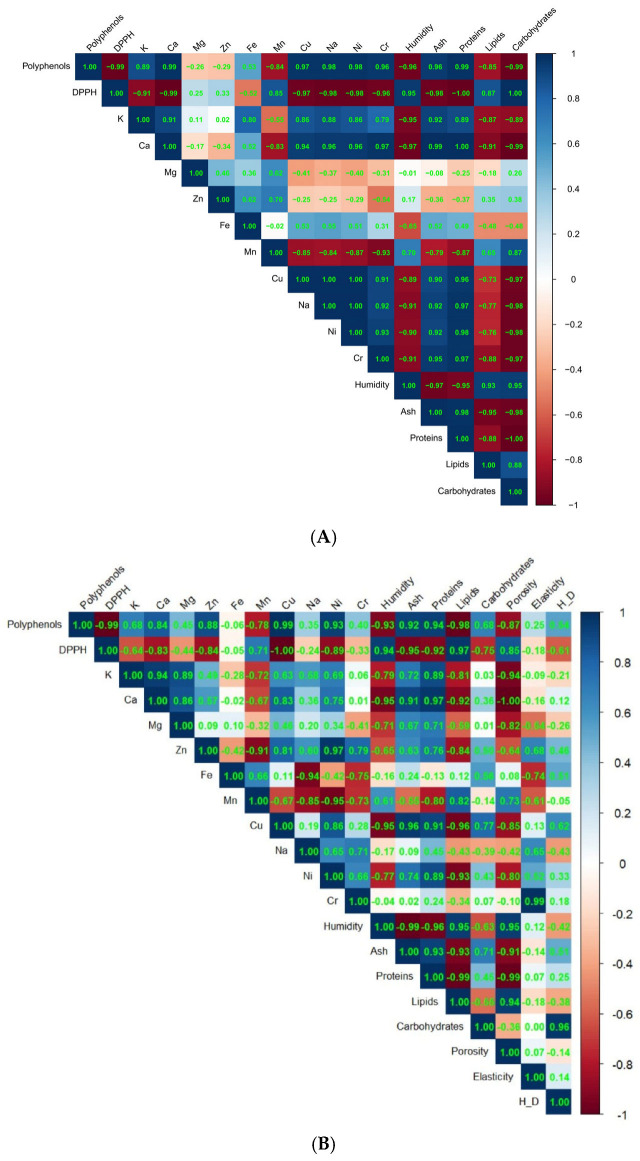
Pearson correlation between physical–chemical, macro and microelements, and phytochemical parameters in composite flours (**A**) with whey powder and in fortified bread (**B**).

In Figure 5, the Pearson correlations between analyzed parameters are presented.

The statistical analysis of the Pearson correlations in samples of fortified flour with whey (Figure 5A) highlighted significant and strong relationships between the polyphenol content, antioxidant activity (DPPH), chemical composition parameters, and mineral content.

Polyphenols exhibited extremely strong correlations with numerous variables, indicating a close association between bioactive compounds and the nutritional composition of the flours. A very strong negative correlation (r = −0.99, *p* = 0.001) was noted between DPPH and polyphenols, confirming that a higher content of polyphenols leads to stronger antioxidant activity (reduction of DPPH). Calcium Ca, Cu, Na, Ni, and Cr displayed very high positive correlations (r between 0.96 and 0.99, *p* < 0.01) relative to polyphenols, suggesting that the addition of whey—rich in minerals—contributes both to an increase in mineral concentration and to the intake of polyphenols, likely through bound protein fractions.

Polyphenols recorded very high positive correlations (r = 0.96–0.99) with proteins and ash, indicating that flours with higher protein and mineral content are also the richest in polyphenols.

The antioxidant activity, expressed by the reduction of DPPH, was also significantly correlated with most variables. Thus, strong negative correlations were recorded with minerals (Ca, Cu, Na, Ni, Cr) and proteins (r up to −1.00, *p* < 0.001), confirming that samples richer in active nutrients (derived from whey) have a greater antioxidant capacity. A very high positive correlation was recorded for antioxidant activity with carbohydrates (r = 1.00, *p* < 0.001), reflecting that samples with higher starch content have lower antioxidant activity. Calcium is one of the most correlated parameters, with significant links to Cu (r = 0.94), Na, Ni, and Cr (r > 0.96), as well as to proteins (r = 1.00) and carbohydrates (r = −0.99). This distribution reflects the balance between protein/mineral content and carbohydrate content in the composition of fortified flours. Copper, an oligoelement found in whey, is closely correlated with Na, Ni (r = 1.00), Cr (r = 0.91), and proteins (r = 0.96), reinforcing the hypothesis of a common origin—the concentration of these elements through the addition of whey proteins.

Proteins and carbohydrates show a perfect negative correlation (r = −1.00), reflecting the inversely proportional nature of the flour composition: an increase in the protein and mineral fraction reduces the proportion of starch. Humidity and lipids have a positive correlation (r = 0.93), suggesting that more hydrated samples better retain lipids—possibly through micro-encapsulation effects or physico-chemical interactions.

The analysis of Pearson correlations in bread samples obtained from wheat flour with added whey concentrate (Figure 5B) highlighted a series of significant and very strong relationships between the biochemical composition, functional parameters, and the content of trace elements. These correlations reflect the complex interactions between nutritional components and the influence of the baking process on them.

An extremely strong negative correlation between total polyphenols and antioxidant activity (r = −0.99, *p* = 0.006) indicates that a higher content of polyphenols is associated with increased antioxidant activity (reduction of DPPH value). This relationship confirms the bioactive potential of whey on the functional qualities of bread [2].

The pairs Polyphenols/(Cu) indicate a very high positive correlation (r = 0.99, *p* = 0.014), suggesting an association between the content of polyphenols and the concentration of this trace element, which may be present in the protein fraction of whey. Between polyphenols and lipids, a negative correlation is recorded (r = −0.98, *p* = 0.018), which may indicate that samples with fewer lipids retain phenolic compounds better or that polyphenols may reduce lipid oxidation [15].

A very high positive correlation (r = 0.97, *p* = 0.031) between calcium and proteins supports the fact that whey supplementation simultaneously increases both the protein and mineral content [1].

DPPH correlated perfectly negatively with copper content (r = −1.00, *p* = 0.002), an element involved in redox reactions with a possible synergistic or catalytic role in antioxidant activity [3]. In contrast, the positive correlation with lipids (r = 0.97, *p* = 0.034) may indicate an increased vulnerability to oxidation in samples richer in fats, thus confirming previously reported results in bakery products [14].

The technological importance presents the correlations between the chemical composition and the physical parameters of bread. A strong negative correlation is observed in the case of calcium and porosity (r = −1.00, *p* = 0.004), indicating an inverse relationship between mineral concentration (especially calcium) and the spongy structure of the bread, which can affect texture. A similar negative correlation (r = −0.99) was recorded between proteins and porosity, confirming the influence of protein content on the gluten network and the porosity of the crumb [14]. The positive correlation (r = 0.99) between Cr and the elasticity of the core indicates a possible indirect functional role of chromium in the formation of the gluten network or in the hydration of starch.

There is also a very high negative correlation (r = −0.99) between lipids and proteins, which may reflect the dilution of the lipid fraction in protein-rich samples derived from whey.

The analysis of correlations in fortified bread samples with whey concentrate highlights a complex network of relationships between biochemical components and the functional parameters of the product. Polyphenols and essential minerals (Cu, Ca, Zn, Cr) are significantly associated with the antioxidant activity and structural properties of the bread, while moisture, porosity, and lipids are negatively influenced by the increase in protein content.

### 3.6. Rheological Analysis of Fortified Bread with Whey Powder

The rheological behavior of the dough was evaluated using Mixolab analysis, which allows for the simultaneous examination of protein and starch transformations under kneading, heating, and cooling conditions.

**Figure 6:**
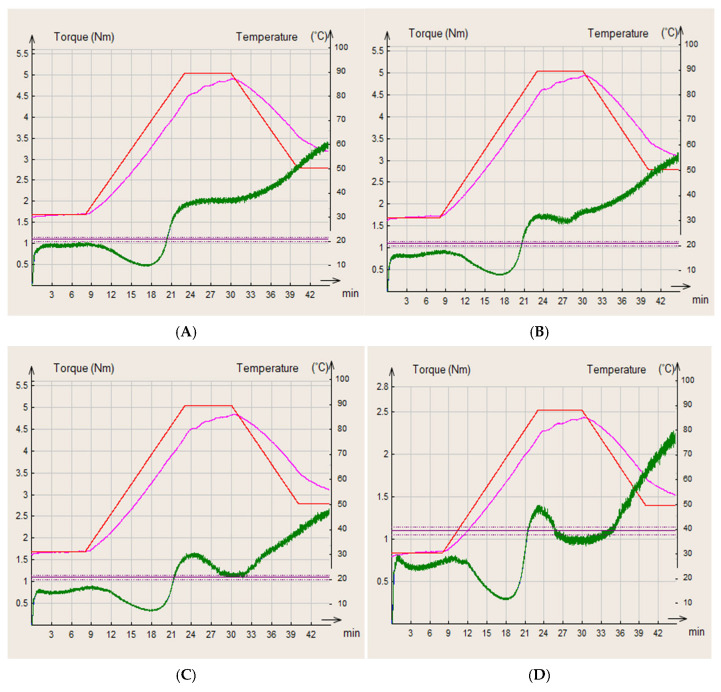
Mixolab rheological profiles of the analyzed flour samples: WF (**A**), WWhF5 (**B**), WWhF10 (**C**), and WWhF15 (**D**). Red line—MIXOLAB temperature (°C), pink line—dough temperature (°C), green line—MIXOLAB curve.

In Figure 6, the MIXOLAB profiles of the control sample (WF) and composite flours (WhWB5, WhWB10, WhWB15) are presented. On the left vertical axis, the torque (Nm) is represented—that is, the resistance of the dough to kneading. On the right axis is the temperature (°C), and on the horizontal axis is time (minutes).

The curves highlight the phases of the process:

C1—Protein stability during kneading (0–8 min, ~30 °C). The initial torque (green) is around 1.5–1.7 Nm, indicating relatively good hydration capacity and gluten formation. If this value is lower than that of the control flour (WF), it means that the addition of WhF dilutes the gluten network.

C2—Weakening of proteins upon heating (up to ~15 min, ~60 °C). The lowest value (minimum value) of the green curve indicates protein degradation (denaturation). A lower C2 point indicates lower protein resistance—often associated with the addition of non-gluten ingredients (such as WhF).

C3—Gelatinization of starch (~25 min, ~90 °C): The torque reaches a maximum (~4.7–5.0 Nm), signaling good starch gelatinization capacity. If this value is lower than that of the control flour, the addition of WhF has diluted the starch, and gelatinization capacity is affected.

C4—Stability of starch at high temperature (~30–34 min). A slight decrease in torque is observed, indicating partial lysis of starch granules or the action of enzymes (amylases). If the value is stable, the starch is more resistant to degradation—a positive aspect for the final texture of the bread.

C5—Starch retrogradation during cooling (~38–42 min). The final increase in torque reflects the ability of the starch to retrograde—influencing the firmness and freshness of the bread. Lower torque in this area may indicate less tendency to stale, due to the presence of WhF proteins that can interfere with starch retrogradation.

The analyzed Mixolab curve suggests that the addition of WhF significantly influences the rheological behavior of the dough (Figure 6). In the initial phase (C1), the relatively moderate torque suggests a good capacity for protein network formation but a diluted potential compared to standard wheat flour, a frequent occurrence with non-gluten additions. The decrease in value C2 indicates a weakening of the protein under the action of temperature, suggesting that the proteins in WhF have lower thermal stability. In the C3 zone, the high level of torque shows that the present starch still has gelatinization capacity but is likely influenced by the additional protein contribution. The C4–C5 zone reflects good thermal stability and moderate retrogradation, which can positively contribute to the texture and freshness retention of the final product.

Protein additives (including those from insects or whey) reduce the stability of the gluten network and can significantly modify the rheological behavior of the dough [30,50]. The presence of alternative proteins can reduce starch retrogradation and thus slow down the staleness of bread [51].

The comparative analysis of the Mixolab curves highlighted clear effects of the WhF addition on the functional properties of the dough. In the initial kneading phase (C1), a high value of the torque for the control dough was observed (~1.7–1.8 Nm), indicating a well-formed protein network specific to wheat gluten. With the increase in the concentration of WhF, the C1 values progressively decreased, reaching ~1.2 Nm at the 15% addition. This decrease reflects a weakening of the gluten network, caused by the partial substitution of gluten proteins with soluble whey proteins, which do not contribute equally to the formation of the elastic gluten network.

Similar results are reported by Kučerová et al. (2021) [50], who observed a decrease in consistency and stability during kneading in doughs with alternative protein additives. In the heating stage (C2), the minimum of the curve was significantly more pronounced in samples with WhF, especially at 10% and 15%, indicating a pronounced denaturation of proteins due to temperature effects. This behavior is associated with the less thermally stable structure of whey proteins compared to gluten. The results confirm data from the literature regarding the behavior of unconventional protein ingredients in complex thermal systems [30]. The starch gelatinization stage (C3) highlighted a clear peak for the control flour (~5 Nm), suggesting a good swelling and gelation capacity of the starch. In contrast, in mixtures with WhF, especially at 15%, this peak was significantly diminished (~2.7–2.8 Nm), reflecting either a lower available starch content or a physicochemical interference of WhF components with the gelatinization process. Delcour and Hoseney (2010) [51] emphasized that the presence of soluble proteins or other functional compounds can negatively affect the gelatinization capacity of starch by limiting water absorption and altering the swelling structure.

In the thermal stability phase of starch (C4), the control dough maintained a stable torque range, while the samples with WhF showed a more pronounced decrease, suggesting either an increased sensitivity to high temperatures or an increased amylolytic activity in the protein mixtures. Ultimately, the cooling stage (C5), associated with starch retrogradation, showed an increase in torque in all samples. In the case of the dough with 15% WhF, the final torque reached values comparable to the control, indicating a preserved or even slightly increased retrogradation capacity. This may have favorable implications for the crumb structure and textural stability of baked goods over time, as controlled retrogradation contributes to maintaining freshness [49].

The data reported by Pořízka et al. (2023) indicated a decrease in water absorption compared to the reference sample simultaneously with the formation of a complex system that hindered hydration, extensibility, and wheat gluten alignment, leading to an increase in dough development time by 5 min with 5% protein whey added in the bread [52].

In conclusion, the addition of WhF significantly influences the rheological properties of the dough. Although it tends to diminish kneading resistance and protein stability, negatively affecting gluten structure and starch gelatinization, these effects are balanced by a high functional potential, especially from a nutritional standpoint.

**Figure 7:**
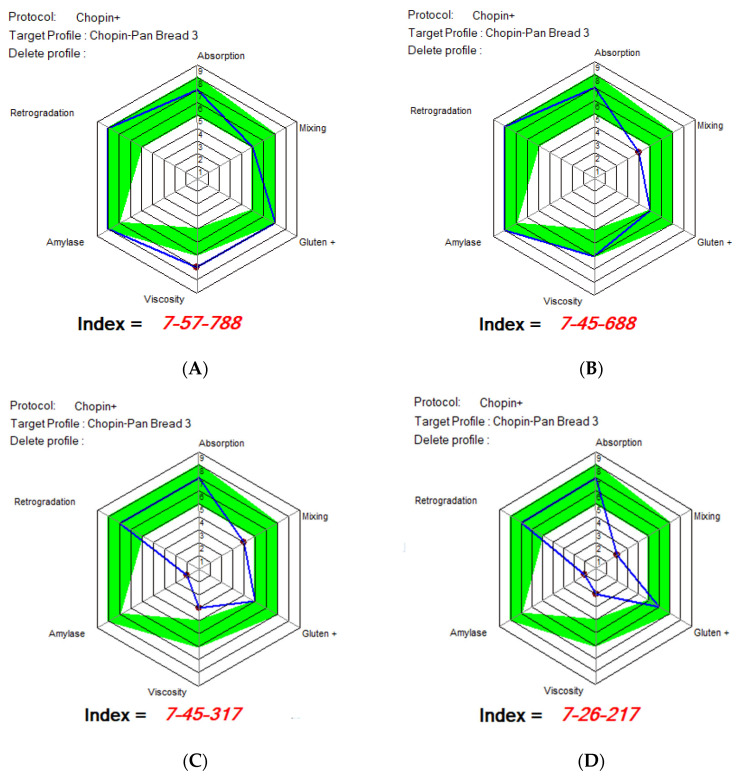
Radar plots rheological profiles of Mixolab: WF (**A**), WWhF5 (**B**), WWhF10 (**C**), and WWhF15 (**D**) Blue line represents the profile of partially substituted wheat flours and green line represents the profile of optimal MIXOLAB parameters for bread-making technology.

The Figure 7 represents the rheological profiles of Mixolab in the form of radar plots (polar charts), according to the Chopin+ protocol, using the reference profile ‘Chopin-Pan Bread 3’ [27]. These charts provide a synthetic view of the functional behavior of flours in six key areas: water absorption, mixing, gluten network, viscosity, amylase activity, and retrogradation.

The four Mixolab graphs correspond to samples with increasing levels of WhF protein flour addition, reflected by the progressive decrease in the Global Baking Index. The index consists of three components: water absorption—gluten quality—starch behavior, each evaluated on a scale of 0–9 (where 9 = optimal performance).

*Water Absorption.* All four samples have a high score (7) for absorption, suggesting a good hydration capacity. This indicates that the addition of WhF does not negatively affect the overall absorption capacity, most likely due to the high protein content and the presence of hydrophilic compounds. Delcour and Hoseney (2010) [51] explain that soluble proteins, such as those in whey, can contribute to water retention even in the absence of gluten network formation.

*Kneading and the gluten network (Mixing and Gluten+).* Samples with WhF additions show a clear decrease in the gluten index, from seven (Figure 7A) to two (Figure 7D). The score for kneading capacity also decreases, from five to one. This trend indicates alteration of the protein network, resulting from the dilution of gluten with non-network-forming proteins. In the specialized literature, this phenomenon is well documented: whey or insect proteins cannot directly replace the elastic structure provided by gluten, affecting the extensibility and cohesion of the dough [30,50].

*Viscosity index.* The viscosity score decreases considerably from Figure 1 to Figure 4, suggesting a reduced ability of the starch to gelatinize and form a coherent mass. This is likely determined by a lower amount of starch in the total formula (due to the addition of WhF) or by the interference of additional proteins with the gelatinization processes. According to Gómez et al. (2003) [53], protein-rich additives can reduce the viscosity and final consistency of the dough by the competitive absorption of water and by limiting starch swelling.

*Amylase activity.* The index for amylase activity is low in all samples (around one to two), which suggests a moderate to low activity of amylases, typical of thermally treated flours or those with additives that have low enzymatic activity. It is possible that WhF does not significantly contribute to amylolytic activity.

*Retrogradation.* The score for retrogradation decreases slightly from the control (~6) to the test with 15% WhF (~3). A reduced retrogradation may indicate greater stability over time of the bread crumbs, delaying the aging process. This aspect is considered favorable in functional baked goods, where extending the freshness duration is desired [49].

The analysis of the Mixolab radar charts confirms the progressive impact of the addition of WhF on rheological behavior. While water absorption remains high, other functional characteristics, such as gluten quality, kneading capacity, and starch viscosity, are negatively impacted. The decrease in the Global Index from 788 to 217 reflects losses in technological performance, especially with the addition of 15% WhF. However, nutritional benefits and the trend toward lower retrogradation may balance these disadvantages in the context of developing functional products. Formulations with up to 5–10% WhF seem to offer an acceptable balance between functionality and nutritional value.

### 3.7. Sensory Evaluation of Fortified Bread with Whey Powder

**Figure 8:**
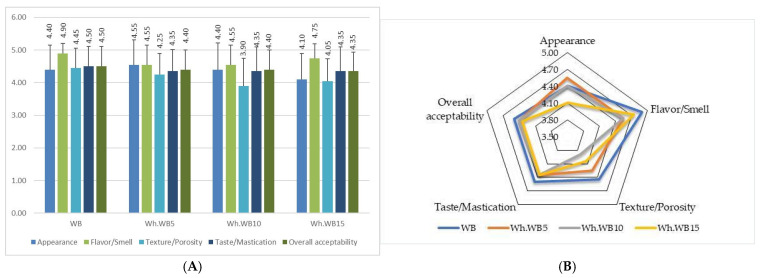
Consumer acceptance of bread with different whey proportions, using a 5-point hedonic scale (n = 20) (**A**) Mean values of appearance, aroma/flavor, texture/porosity, taste/chewiness, and overall acceptability. (**B**) Sensory chart based on bread attributes.

Sensory analysis of whey bread samples was conducted to determine consumer acceptability based on various attributes such as appearance, aroma/flavor, texture/porosity, taste/chewiness, and overall acceptability. Acceptance (affective) testing refers to the measurement of liking or preference and is a valuable and necessary component in determining the quality of a food product. In Figure 8, the sensory analysis of bread fortified with whey powder is presented.

As shown in Figure 8, the white bread (WB) sample generally obtained higher scores for all sensory attributes analyzed, with close average scores ranging from 4.50 to 4.90. Appearance, flavor, and overall acceptability were all highly rated, falling in the high acceptability range (4.00–5.00). The addition of whey powder at concentrations of 5%, 10% and 15% slightly influenced the sensory scores, with a decrease as the concentration increased. Regarding the aspect, the WhWB5 sample records a higher score (4.55) compared to the control sample (4.40), but there is a considerable reduction in the other attributes: flavor 4.55 compared to 4.90 for WB, 4.25 for porosity compared to 4.45, 4.35 for chewability compared to 4.50 (WB), and general acceptability of 4.40 points compared to the maximum value of 4.50 awarded to bread without whey. The addition of 10% whey in bread does not differ significantly from the WhWB5 sample, with slight differences recorded in texture and appearance. A significant deterioration in sensory parameters is observed with the addition of 15% whey for all analyzed parameters, especially for the taste attribute that becomes sour, with the index of this attribute on the hedonic scale being 4.05.

Similar studies were carried out by Pořízka J et al. in 2023 [52], who investigated the physicochemical and sensory properties of bread with whey protein isolates. Bread with a high whey protein substitution was associated with reduced overall acceptability due to a sour taste of the studied samples.

In conclusion, the addition of whey powder to bread influences the sensory acceptability of the product, and this influence becomes more evident as the concentration increases. White bread (WB) obtained the highest scores in all sensory attributes. The addition of 5–10% whey powder in bread had minimal impact and maintained a good level of acceptability, but higher concentrations led to significant decreases in scores.

### 3.8. Spectroscopy Analysis (FTIR) of Fortified Flours with Whey Powder

**Figure 9:**
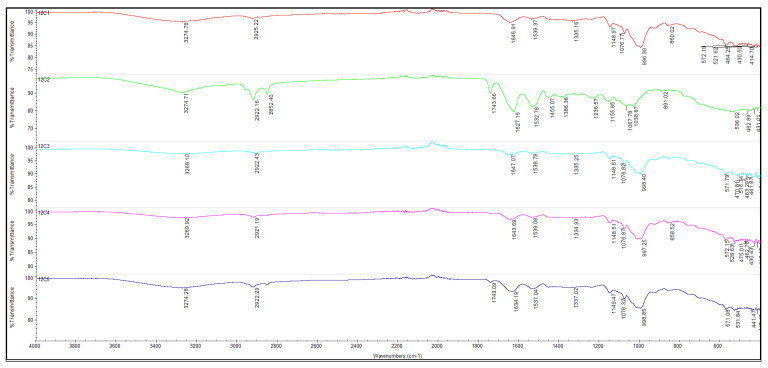
FTIR spectrum of fortified flours with whey powder: WF (red line), WhF (green line), WhWF5 (light blue line), WhWF10 (pink line), and WhWF15 (dark blue line), spectral range of 4000–400 cm^−1^, 32 scans at 4 cm^−1^ resolution.

Fourier transform infrared spectroscopy (FTIR) was used to investigate the functional composition of wheat flour (WF), whey powder (WhF), and their mixtures (WhWF5, WhWF10, WhWF15), in order to highlight the molecular interactions and structural changes occurring due to the addition of whey (Figure 9). Subsequently, the spectrum analysis focused on several characteristic regions: around 3300 cm^−1^, where bands associated with O-H bonds appear; around 1650 cm^−1^, corresponding to bound starch; and in the 1047/1022 cm^−1^ range, useful for highlighting the degree of crystalline versus amorphous order in the structure.

The FTIR spectrum of wheat flour exhibits characteristic bands for its major components—starch and proteins. A broad band around 3270 cm^−1^ is noted, attributed to the O–H stretching vibrations of hydroxyl groups in polysaccharides and bound water. Another important band is found in the region of 2925 cm^−1^, specific to C–H vibrations of methylene linkages. In the fingerprint region, bands appear around 1635 cm^−1^ (corresponding to C=O linkages of amides—amide I) and 1530 cm^−1^ (amide II), indicating the presence of proteins (especially gluten). Signals in the 1150–1000 cm^−1^ range reflect C–O–C and C–O vibrations of starch.

The FTIR spectrum of whey powder (WhF) highlights a higher intensity of bands corresponding to proteins. The band around 1640 cm^−1^ (amide I) is more prominent, suggesting a high protein concentration, particularly soluble proteins such as β-lactoglobulin and α-lactalbumin. The band at 1545 cm^−1^ (amide II) is also pronounced. Another characteristic signal of whey appears in the range of 1400–1450 cm^−1^, associated with carboxyl groups and C–N. The range of 1050–1000 cm^−1^ remains visible, but is dominated by protein contributions rather than those of carbohydrates.

Wheat flour mixtures—whey powder (WhWF10, WhWF15). The progressive addition of whey powder to wheat flour leads to gradual changes in the FTIR spectra, reflecting the increasing proportion of whey proteins in the starch–protein matrix of the wheat flour.

A slight intensification of the amide I and II bands (~1640 cm^−1^ and ~1540 cm^−1^) is observed in the WhWF5 sample, indicating an increase in protein content. Minor changes also occur in the 1400–1450 cm^−1^ region, signaling contributions from the carboxyl groups of amino acids in whey.

When 10% whey powder was added (WhWF10), the characteristic bands of proteins became more pronounced, especially amide I (~1638 cm^−1^). Additionally, absorption increases in the 1400–1200 cm^−1^ region, suggesting interactions between the polysaccharides in the flour and the protein components in the whey. The 1000–1100 cm^−1^ area remains present but shows a modified spectral profile, indicating possible interactions or supramolecular structures [54].

The spectrum of the WhWF15 sample clearly highlights the dominance of soluble proteins. The amide I band becomes broad and well-defined, while the amide II signal gains intensity. The prominent presence of the band at 1450 cm^−1^ suggests a greater amount of COO^−^ groups. These changes indicate not only a quantitative increase in whey but also possible interactions between whey proteins and the gluten in flour [55].

According to the literature, the characteristic FTIR bands of whey proteins are found in the regions 1700–1500 cm^−1^, while those of starch and carbohydrates appear in 1200–900 cm^−1^ [56]. Studies have shown that in mixtures with flour, whey proteins can form alternative protein networks, affecting rheological behavior and water retention capacity [57]. The increase in the intensity of the amide bands, along with changes in the hydroxyl region (3200–3400 cm^−1^), confirms these interactions as well in the studied samples [58].

### 3.9. Microscopic Evaluation of Composite Flours with Whey Powder

**Figure 10:**
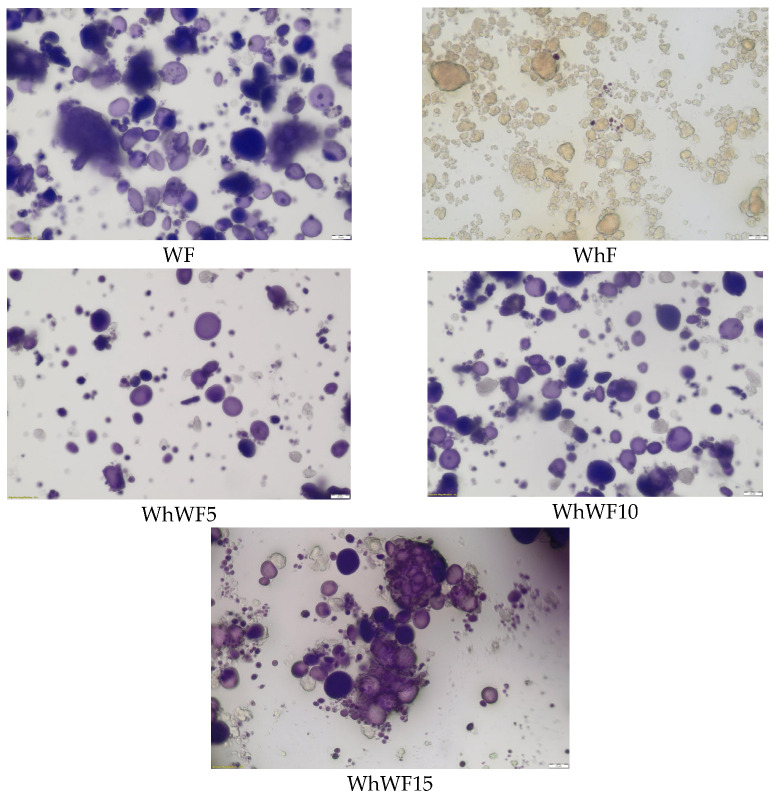
The microscopic evaluation of composite flours with whey powder (40× objective).

In Figure 10, the microscopic evaluation of composite flours with whey powder is presented.

The microscopic analysis was conducted to evaluate the morphological changes in the structure of wheat flour particles following the addition of whey powder in proportions of 5%, 10%, and 15%. The images were obtained using a standard optical microscope, allowing for the observation of surface characteristics and particle aggregation. The control sample, represented by 100% wheat flour, exhibited a uniform structure, with well-defined granules typical of refined flour. The particles displayed regular edges, with homogeneous sizes, typical characteristics of wheat starch. These observations are consistent with the literature, which describes wheat starch granules as polyhedral and well-defined in refined preparations [59].

The addition of 5% whey powder resulted in a slight aggregation of particles, leading to the formation of larger conglomerates and a less uniform distribution of granules. This modification can be attributed to the high protein content of the whey powder, which acts as a binding agent between particles, a phenomenon also reported in other studies on flour and milk protein blends [60].

At a concentration of 10% whey powder, significant changes were observed in the matrix structure, with an increase in the degree of aggregation and a reduction in the clarity of individual granules. These changes suggest a more intense interaction between the whey proteins and wheat starch, which may lead to functional modifications of the rheological properties of the flour [61].

In the case of the sample with 15% whey powder, the aggregation was even more pronounced, with the formation of compact masses and a visible reduction in the individuality of the granules. This dense structure can significantly affect the hydration of flour, the dough formation capacity, and behavior during baking. The literature suggests that a high whey protein content can negatively influence the technological properties of the dough due to interference with the gluten network [62].

Microscopic images confirm the significant influence of whey powder on the microstructure of wheat flour. As the concentration of whey increases, a transition occurs from a clearly granular structure to a compact matrix, with potential implications for subsequent technological processes. These data highlight the importance of optimizing the level of functional ingredient additions to maintain the desired properties of baked goods.

### 3.10. Small—Wide Angle X-Ray Scattering (SAXS/WAXS) of Fortified Flours with Whey Powder

**Figure 11:**
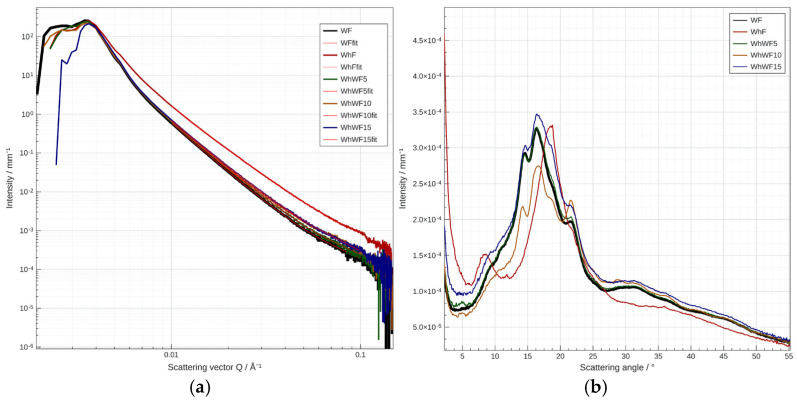
(**a**,**b**) The analysis of Small -Wide Angle X-ray Scattering (SAXS/WAXS) WF (black line), WhF (red line), WhWF5 (green line), WhWF10 (orange line), and WhWF15 (blue line).

Small—Wide Angle X-ray Scattering provides valuable information about the crystalline organization of the starch and protein constituents in wheat flour, affected by the addition of whey powder (Table 6, Figure 11a,b). SAXS/WAXS signals are predominantly associated with the crystalline phases of starch (type A or B), but the interaction with the proteins in whey can significantly modify the intensity and width of the characteristic bands. From SAXS region data, the wheat flour (WF) has a larger average medium structure (904 nm median, 88 nm equivalent surface) compared to whey powder (WhF), which has smaller structural particles (878 nm, 57 nm). This indicates that whey has a more compact structure or forms smaller aggregates. At 5% whey (WhWF5), the median size slightly decreases (897 nm), and the surface size is 78 nm; a slight reduction compared to 100% wheat flour was observed. At 10% whey (WhWF10), an even greater reduction was observed (891 nm, 70 nm), while at 15% whey (WhWF15), a slight increase compared to 10% (895 nm, 74 nm) was presented. This behavior suggests that the addition of whey gradually reduces the average size of the structures present in the mixture. Fortifying with up to 10% whey has a compressive effect on the structure; above this threshold (15%), the structure begins to increase slightly, possibly due to aggregation or interaction between the whey proteins and the starch in the wheat flour, a phenomenon observed also in the case of microscopic analysis. Regarding goodness of fit, all values are close to 1, indicating good and reliable fit models.

Recent studies indicate that whey proteins can induce significant changes in the crystalline structure of starch. Zhu et al. (2020) demonstrated that the addition of whey protein isolate (WPI) to wheat starch results in an increase in relative crystallinity, by promoting the reorganization of amylopectin chains [63]. In similar cracker-type formulations, Madhav et al. (2025) observed a slight reduction in crystallinity when the flour was fortified with proteins (soy, pea, whey), which is a consequence of the interference of proteins with the ordered structure of starch [64].

The fortification of wheat flour with whey influences the internal structure of the material. Whey, having a finer structure, reduces the average size of aggregates in flour to a certain level (10%), after which complex interactions may lead to a slight structural increase. This can have implications for texture, solubility, and functional behavior in food applications.

The analysis of WAXS provides a detailed view of the degree of crystalline order in the fortified wheat flour samples with whey powder. These structures are critical in determining the physicochemical and functional properties of starch–protein blends. The graph presented in Figure 11b highlights significant changes in the intensity and shape of the diffraction peaks for the five analyzed samples: pure wheat flour (WF), whey powder (WhF), and blends with 5%, 10%, and 15% whey (WhWF5, WhWF10, WhWF15).

In the amorphous region (2θ = 5–10°), the high intensity observed for sample WhF reflects the presence of dominant amorphous fractions in whey, such as lactalbumin and β-lactoglobulin. These components form disordered aggregates, with minimal contributions to coherent diffraction. With the fortification of flour with whey, a pronounced decrease in intensity is observed in this range, indicating an interference of whey with the ordered networks of starch, particularly through the inhibition of its retrogradation, similar to the observations reported by Zhu et al. (2020) [63].

In the crystallinity region of starch (2θ = 13–24°), wheat flour (WF) shows pronounced peaks around the values of 15°, 17°, and 23°, corresponding to the crystalline structure type A, typical of cereal starch [65]. The addition of whey leads to a flattening and a significant decrease in these peaks, especially in samples WhWF5 and WhWF10, where crystallinity is minimal. This phenomenon is attributed to the electrostatic interaction between the soluble whey proteins and the side chains of amylopectin, which affects the formation of semicrystalline networks, according to the conclusions of Madhav et al. (2025) [64]. The behavior of sample WhWF15 is interesting: although the intensity of the peaks is still lower than the control, a slight recovery of structural ordering is observed, possibly due to the formation of new proteo-starch complexes, reorganized into a more stable semicrystalline structure. This effect of ‘protein-induced recrystallization’ has also been described by Li et al. (2023) [66] in starch–vegetal protein systems.

At the high-angle region (2θ > 25°), the samples do not show significant diffraction peaks, indicating a lack of formation of additional crystalline phases or higher-order molecular rearrangements. Moreover, the relatively flat general background confirms the predominantly semicrystalline nature of the analyzed systems.

The reduction in relative crystallinity through whey fortification can have important implications for the physical–functional properties of the final product. The decrease in starch ordering is associated with increased solubility and digestibility, reduced resistance to retrogradation (hardening over time), and changes in gelatinization behavior. Thus, the addition of 10% whey seems to represent the critical point of imbalance between the crystalline and amorphous structures, while at 15% there is a tendency for the reconfiguration of a stabilized protein–starch complex structure.

WAXS results indicate a progressive decrease in the crystalline order of starch by fortifying the flour with whey flour, with a clear minimum observed at an addition of 10%. The structural effects can be attributed to the specific interaction between whey proteins and the polysaccharide chains in starch. At a higher concentration (15%), the proteins seem to induce a structural reorganization that partially compensates for the earlier losses in crystallinity.

## 4. Conclusions

In conclusion, the obtained data highlight the positive impact of adding WhF on the nutritional value of composite flours and bakery products, particularly through the increase in protein and mineral content, along with a reduction in carbohydrate content. The correlations observed in the samples of whey-fortified flour indicate a strong interdependence between the mineral composition, protein content, and antioxidant activity. Fortifying wheat flour with whey powder modifies the crystalline structure of starch and the behavior of proteins during the technological process, influencing the texture, gelatinization, digestibility, and stability of the final product, with significant implications for the design of functional food formulations.

Based on all the data provided from physicochemical, sensory, rheological, spectroscopic, and microscopic analysis of bread fortified with whey powder, it can be concluded that formulations with up to 5–10% WhF seem to provide an acceptable balance between functionality and the sensory and nutritional values. These results support dairy processors in the purpose of valorizing the whey by-product in the context of the circular economy. Considering that the results obtained are under laboratory/pilot conditions, validation on an industrial scale is necessary to confirm the behavior in complex technological processes. Future research on long-term stability analysis, namely monitoring textural, structural, and sensory changes in bread fortified with whey powder during storage, including the impact on shelf life, is also recommended.

## Figures and Tables

**Table 1:** Recipes for fortified breads with whey powder.

	Whey Powder (g)	Wheat Flour Type 550 (g)	Yeast (g)	Salt (g)	Sugar (g)	Water (mL)
WB	-	100	3	2	1	50
WhWB5	5	95	3	2	1	50
WhWB10	10	90	3	2	1	50
WhWB15	15	85	3	2	1	50

**Table 2:** Proximate composition of fortified flours and breads.

Samples	Nutritional Characteristics				
	Humidity	Ash	Proteins	Lipids	Carbohydrates
	(%)	(%)	(%)	(%)	(g/100 g)
Composite flours					
WF	12.70 ± 0.15 ^a^	0.59 ± 0.04 ^e^	11.26 ± 0.02 ^a^	1.35 ± 0.02 ^a^	74.11 ± 0.11 ^a^
WhF	9.41 ± 0.14 ^d^	3.01 ± 0.02 ^a^	85.85 ± 0.03 ^a^	0.53 ± 0.02 ^e^	1.21 ± 0.11 ^e^
WhWF5	12.11 ± 0.21 ^b^	1.25 ± 0.04 ^d^	25.24 ± 0.06 ^d^	0.97 ± 0.02 ^b^	60.44 ± 0.28 ^b^
WhWF10	11.59 ± 0.01 ^c^	1.64 ± 0.03 ^c^	31.77 ± 0.02 ^c^	0.84 ± 0.01 ^c^	54.17 ± 0.02 ^c^
WhWF15	10.60 ± 0.26 ^d^	1.77 ± 0.02 ^b^	37.77 ± 0.10 ^b^	0.76 ± 0.01 ^d^	49.10 ± 0.18 ^d^
Breads					
WB	29.37 ± 0.10 ^a^	1.33 ± 0.02 ^b^	14.12 ± 0.04 ^d^	6.68 ± 0.02 ^a^	48.49 ± 0.08 ^a^
WhWB5	28.32 ± 0.24 ^b^	1.48 ± 0.26 ^a,b^	16.64 ± 0.03 ^c^	5.46 ± 0.04 ^b^	47.96 ± 0.29 ^b^
WhWB10	27.43 ± 0.03 ^c^	1.88 ± 0.18 ^a^	17.01 ± 0.03 ^b^	4.96 ± 0.03 ^c^	47.83 ± 0.19 ^b^
WhWB15	27.03 ± 0.06 ^d^	2.40 ± 0.16 ^a^	18.89 ± 0.03 ^a^	3.47 ± 0.01 ^d^	48.21 ± 0.15 ^a^

The values are expressed as mean values ± standard deviations of all measurements; data sharing different letters in the same column are significantly different (*p* < 0.05), according to the Duncan test.

**Table 3:** Macro and microelements content of composite flours and breads fortified with whey powder.

Samples	Macro and Microelements Content (mg/kg)									
	K	Ca	Mg	Zn	Fe	Mn	Cu	Na	Ni	Cr
Composite flours										
WF	1388.26 ± 70 ^d^	337.58 ± 0.50 ^e^	532.5 ± 1.00 ^d^	11.18 ± 0.80 ^b^	11.34 ± 0.90 ^c^	8.83 ± 0.56 ^a^	2.64 ± 0.45 ^c^	40.85 ± 0.37 ^d^	0.88 ± 0.08 ^d^	0.60 ± 0.15 ^a^
WhF	2264.54 ± 20 ^a^	802.69 ± 0.50 ^a^	510.74 ± 0.40 ^e^	7.82 ± 0.60 ^d^	13.46 ± 0.60 ^a,b^	0.76 ± 0.19 ^c^	19.33 ± 0.60 ^a^	111.1 ± 0.67 ^a^	10.9 ± 0.38 ^a^	0.84 ± 0.11 ^a^
WhWF5	1320.78 ± 0.70 ^e^	438.98 ± 0.40 ^d^	563.88 ± 0.10 ^c^	6.52 ± 0.30 ^e^	7.65 ± 0.20 ^d^	5.50 ± 0.17 ^b^	1.55 ± 0.38 ^d^	38.03 ± 0.38 ^e^	0.83 ± 0.09 ^d^	0.69 ± 0.07 ^a^
WhWF10	1809.25 ± 10 ^c^	480.10 ± 0.63 ^c^	671.47 ± 0.80 ^b^	9.56 ± 0.50 ^c^	12.26 ± 0.60 ^b,c^	8.38 ± 0.44 ^a^	3.45 ± 0.30 ^c^	48.66 ± 0.57 ^c^	1.71 ± 0.08 ^c^	0.66 ± 0.09 ^a^
WhWF15	1906.19 ± 0.70 ^ab^	544.78 ± 0.45 ^b^	702.84 ± 0.70 ^a^	11.31 ± 0.60 ^a^	14.23 ± 0.60 ^a^	8.55 ± 0.30 ^a^	5.41 ± 0.60 ^b^	55.56 ± 0.36 ^b^	2.83 ± 0.33 ^b^	0.69 ± 0.17 ^a^
Fortified Breads										
WB	1207.06 ± 0.90 ^d^	360.28 ± 0.60 ^d^	530.84 ± 0.40 ^d^	10.87 ± 0.70 ^a^	8.49 ± 0.20 ^b^	5.75 ± 0.65 ^a^	1.52 ± 0.32 ^c^	209.18 ± 0.57 ^c^	0.55 ± 0.28 ^b^	0.71 ± 0.26 ^a^
WhWB5	1531.09 ± 50 ^b^	464.07 ± 0.30 ^c^	581.75 ± 0.30 ^a^	9.91 ± 0.10 ^b^	7.78 ± 0.30 ^c^	5.32 ± 0.36 ^a,b^	1.74 ± 0.17 ^b,c^	221.08 ± 0.34 ^b^	0.85 ± 0.10 ^b^	0.68 ± 0.18 ^a^
WhWB10	1431.19 ± 0.50 ^c^	475.18 ± 0.60 ^b^	578.27 ± 0.60 ^b^	11.45 ± 0.50 ^a^	10.09 ± 0.20 ^a^	5.74 ± 0.30 ^a^	2.38 ± 0.55 ^a,b^	201.19 ± 0.61 ^d^	0.78 ± 0.20 ^b^	0.64 ± 0.14 ^a^
WhWB15	1542.59 ± 0.70 ^a^	505.13 ± 0.50 ^a^	568.08 ± 0.70 ^c^	16.68 ± 0.40 ^c^	7.87 ± 0.40 ^b,c^	4.73 ± 0.50 ^b^	2.87 ± 0.19 ^a^	222.85 ± 0.88 ^a^	1.72 ± 0.15 ^a^	0.75 ± 0.16 ^a^

The values are expressed as mean values ± standard deviations of all measurements; data sharing different letters in the same column are significantly different (*p* < 0.05), according to the Duncan test.

**Table 4:** The volume, porosity, elasticity, and height/diameter (H/D) ratio of fortified breads with whey powder.

Indices	M.U.	WB	WhWB5	WhWB10	WhWB15
Porosity	%	77.87 ± 0.01 ^a^	73.22 ± 0.02 ^b^	72.89 ± 0.04 ^c^	70.91 ± 0.01 ^d^
Elasticity	%	90.00 ± 6.01 ^a^	86.53 ± 2.45 ^a,b^	82.96 ± 0.50 ^b^	80.95 ± 3.80 ^b^
H/D	-	0.945 ± 0.003 ^b^	0.885 ± 0.002 ^c^	0.965 ± 0.04 ^a^	0.966 ± 0.002 ^a^

The values are expressed as mean values ± standard deviations of all measurements; data sharing different letters in the same row are significantly different (*p* < 0.05), according to the Duncan test.

**Table 5:** The phytochemical profile of fortified flours and breads with whey powder.

Samples	Total Polyphenols Content (mg/100 g)	Antioxidant Activity, DPPH (%)
Composite flours		
WF	184.98 ± 1.14 ^e^	28.26 ± 0.44 ^a^
WhF	532.56 ± 2.02 ^a^	20.54 ± 0.21 ^e^
WhWF5	230.46 ± 2.87 ^d^	27.19 ± 0.11 ^b^
WhWF10	237.37 ± 2.63 ^c^	26.27 ± 0.15 ^c^
WhWF15	323.38 ± 2.89 ^b^	25.57 ± 0.15 ^d^
Fortified Breads		
WB	229.29 ± 1.72 ^d^	27.25 ± 0.04 ^a^
WhWB5	254.13 ± 1.15 ^c^	26.75 ± 0.01 ^b^
WhWB10	289.13 ± 0.64 ^b^	25.47 ± 0.01 ^c^
WhWB15	348.53 ± 0.97 ^a^	24.18 ± 0.01 ^d^

The values are expressed as mean values ± standard deviations of all measurements; data sharing different letters in the same column are significantly different (*p* < 0.05), according to the Duncan test.

**Table 6:** SAXS parameters of composite flours with whey powder.

Sample	Median of the Distribution, nm	Surface Equivalent Mean Size, nm	Goodness of Fit
WF	904	88	1.00
WhF	878	57	1.02
WhWF5	897	78	1.02
WhWF10	891	70	1.00
WhWF15	895	74	1.00

## Data Availability

The original contributions presented in this study are included in the article. Further inquiries can be directed to the corresponding authors.
